# Expression Level and Significance of Tim-3 in CD4^+^ T Lymphocytes in Peripheral Blood of Patients with Coronary Heart Disease

**DOI:** 10.21470/1678-9741-2020-0509

**Published:** 2022

**Authors:** Jian Zhang, Feng Zhan, Huiling Liu

**Affiliations:** 1 Department of Clinical Laboratory, ChangZhou Tumor Hospital affiliated to Soochow University, ChangZhou, China.

**Keywords:** Coronary Disease. CD4-Positive T-Lymphocytes. Interleukin-7. IL7 protein, human. Severity of Illness Index. Mucin-3. Flow Cytometry. Serum

## Abstract

**Objective:**

To investigate the expression level and significance of T cell immunoglobulin and mucin-domain containing molecules-3 (Tim-3) and interleukin-7 (IL-7) in CD4+ T lymphocytes in peripheral blood of patients with coronary heart disease (CHD).

**Methods:**

75 patients with CHD treated at our hospital were selected and classified as mild group (25 cases), moderate group (25 cases) and severe group (25 cases), according to the severity of illness. Twenty-five healthy volunteers who underwent a physical examination at our hospital during the same period were selected as the control group. The expression level of Tim-3 in CD4^+^ T lymphocytes in peripheral blood of patients in four groups was detected by flow cytometry and quantitative real-time reverse transcription polymerase chain reaction (qRT-PCR). The expression level of IL-7 in peripheral blood serum was measured by enzyme-linked immunosorbent assay (ELISA). Correlation analyses of Tim-3 and IL-7, Tim-3 and disease severity and IL-7 and disease severity were performed, respectively.

**Results:**

Flow cytometry and qRT-PCR demonstrated that the expression of Tim-3 in CD4^+^ T lymphocytes in peripheral blood of patients with CHD increased with the aggravation of the disease. ELISA showed that the tendency of IL-7 expression in peripheral blood serum was consistent with the expression of Tim-3, and the expression of Tim-3 had a positive correlation with IL-7. The expression levels of both Tim-3 and IL-7 were positively correlated with the Gensini score. Conclusion: The expression of Tim-3 and IL-7 in peripheral blood of patients with CHD was upregulated and increased with the aggravation of CHD.

**Table:** 

Abbreviations, acronyms & symbols	
CHD	= Coronary heart disease
ELISA	= Enzyme-linked immunosorbent assay
GAPDH	= Glyceraldehyde 3-phosphate dehydrogenase
IL-7	= Interleukin-7
VC	= Natural killer
PCR	= Polymerase chain reaction
qRT-PCR	= Quantitative real-time reverse transcription polymerase chain reaction
SD	= Standard deviation
SPSS	= Statistical Package for the Social Sciences
Tim-3	= T cell immunoglobulin and mucin domain containing molecules 3

## INTRODUCTION

In recent years, the incidence, disability rate and mortality from coronary heart disease (CHD) have increased due to the faster pace of life ^[1]^, imbalanced dietary pattern ^[2]^, smoking and alcohol consumption ^[3,4]^ and other factors, which seriously threaten human health. Inflammation is an important factor for both the progression of coronary disease and the instability of coronary plaques ^[5,6]^. 

T-cell immunoglobulin and mucin-domain containing molecules-3 (Tim-3), a marker of Th1 cells, plays an important regulatory role in immunoregulation and tolerance. Tim-3 has been demonstrated to regulate a range of inflammatory diseases through multiple signaling pathways ^[7,8]^. In addition, Tim-3, which is highly expressed in natural killer (NK) cells, can serve as a potential marker of disease progression in atherosclerosis ^[9]^. However, the expression and role of Tim-3 in CD4^+^ T lymphocytes in peripheral blood of patients with CHD are unknown. Interleukin-7 (IL-7) is also closely related to the occurrence and development of CHD ^[10]^, and has been demonstrated to induce the expression of Tim-3 on human T cells ^[11]^. Therefore, in this study, we detected the expression of Tim-3 in CD4^+^ T lymphocytes in peripheral blood and the expression of IL-7 in peripheral blood serum of patients with CHD, to analyze the effect of Tim-3 on the progression of CHD and its possible mechanism.

## METHODS

### General Materials

A total of 75 patients with CHD who received treatment at our hospital from January 2018 to December 2019 were selected, as well as 25 healthy volunteers who underwent physical examination at our hospital during the same period. All study subjects have understood the content of the topic under study and signed the informed consent form.

### Selection Criteria

Inclusion criteria were: patients diagnosed with CHD by coronary angiography; patients who met the diagnostic criteria of the World Health Organization for CHD; patients with no history of allergy to contrast media; patients without contraindications to statins; patients whose blood routine, liver and kidney function indexes were normal.

Exclusion criteria were: patients with malignant tumors or infectious diseases; patients with cerebrovascular diseases, autoimmune diseases and diabetes; patients allergic to statins; patients with valvular heart disease; patients with liver and kidney dysfunction.

### Evaluation Criteria for The Degree of Coronary Lesion

The degree of coronary lesion in patients was systematically evaluated according to the Gensini scoring system. According to the degree of stenosis observed by coronary angiography, the single lesion score was as follows: ≤25% was scored as 1 point, 25-50% as 2 points, 50-75% as 4 points, 75-90% as 8 points, 90-99% as 16 points, and 100% as 32 points. The score of the lesion site was obtained by the score of a single lesion × corresponding coefficient, and the calculation method was as follows: left main coronary artery × 5, position of coronary circumflex ostium × 3.5, proximal coronary circumflex artery × 2.5, proximal anterior descending coronary artery × 2.5, middle anterior descending coronary artery × 1.5, distal coronary artery and first diagonal branch × 1, distal coronary circumflex artery × 1, left coronary artery × 0.5, and other branches × 1. The score sum was the total Gensini score: 0-30 points were considered mild, 30-60 points were considered moderate, and more than 60 points were considered severe disease.

### Detection Index

Instruments and reagents: CD4^+^ T lymphocyte isolation kit was purchased from Miltenyi, Germany. Antibodies used for flow cytometry, including anti-CD4^+^-APC Cy7 and anti-Tim-3-PE Texas Red, were purchased from BD, USA. IL-7 ELISA kit was purchased from Solarbio (Art. No.: SEKH-0015). Flow cytometry was a product of BD, USA.

### Collection and Processing of Peripheral Blood

In the morning, after 8 hours of fasting, 10 ml of peripheral venous blood samples were collected from all study subjects in anticoagulant tubes. Then the peripheral venous blood was isolated and purified using a CD4^+^ T lymphocyte isolation kit (Miltenyi, Germany) according to the instructions, and was used subsequently for the detection of Tim-3 by flow cytometry and quantitative real-time reverse transcription polymerase chain reaction (qRT-PCR). In addition, 5 ml of peripheral venous blood samples were collected in non-anticoagulant tubes. After standing and separation, the upper serum was collected and cryopreserved for subsequent ELISA assay.

### Flow Cytometry

The isolated and purified CD4^+^ T lymphocytes were transferred to a flow cytometry tube. After washing with equilibrium buffer, CD4^+^-APC Cy7 (BD, USA) and anti-Tim-3-PE Texas Red (BD, USA) were added to incubate at 4 ºC in the dark for 30 min. After washing again with equilibrium buffer, 2% paraformaldehyde (prepared by PBS) was added to fix before it was loaded on the machine for detection.

### qRT-PCR

Total RNA was extracted using TRIzol (Invitrogen, Carlsbad, CA, USA). Reverse transcription of RNA into cDNA was performed using a reverse transcription kit (TaKaRa, Tokyo, Japan), and all procedures were performed according to the kit instructions. Expression of genes was detected using a LightCycler 480 (Roche, Indianapolis, IN, USA) quantitative fluorescence polymerase chain reaction (PCR) instrument, and reaction conditions were performed according to the operating instructions of the quantitative fluorescence PCR kit (SYBR Green Mix, Roche Diagnostics, Indianapolis, IN). Thermal cycling parameters were as follows: first 95 ºC for 10 s, followed by 45 cycles of 95 ºC for 5 s, 60 ºC for 10 s, and 72 ºC for 10 s, and the final 72 ºC were extended for 5 min. Three replicates were set up for each reaction of quantitative PCR. Glyceraldehyde 3-phosphate dehydrogenase (GAPDH) was used as an internal reference. The 2^− ΔΔCt^ method was used for data analysis. Gene primer sequences are shown in Table 1.

**Table 1:** Primer sequences used in this study.

Primer name	Sequences (5'-3')
Tim-3-F	CTGCTGCTACTACTTACAAGGTC
Tim-3-R	GCAGGGCAGATAGGCATTCT
GAPDH-F	GCAAGGATGCTGGCGTAATG
GAPDH-R	TACGCGTAGGGGTTTGACAC

GAPHD=glyceraldehyde 3-phosphate dehydrogenase

### ELISA Assay

The level of IL-7 in peripheral blood serum was detected using an IL-7 ELISA kit (SEKH-0015, Solarbio, Beijing, China) in strict accordance with the instructions of the kit.

### Statistical Processing

The experimental results were statistically analyzed using SPSS 19.0 software. The means of each group were expressed as mean±standard deviation (SD). One-way analysis of variance was used when multiple groups of data were compared. Correlation analysis was performed using the Pearson correlation test. A *P*<0.05 was considered statistically significant.

RESULTS

### Tim-3 Was Upregulated in CD4+ T Cells in Peripheral Blood of Patients with Coronary Heart Disease

**Fig. 1:**
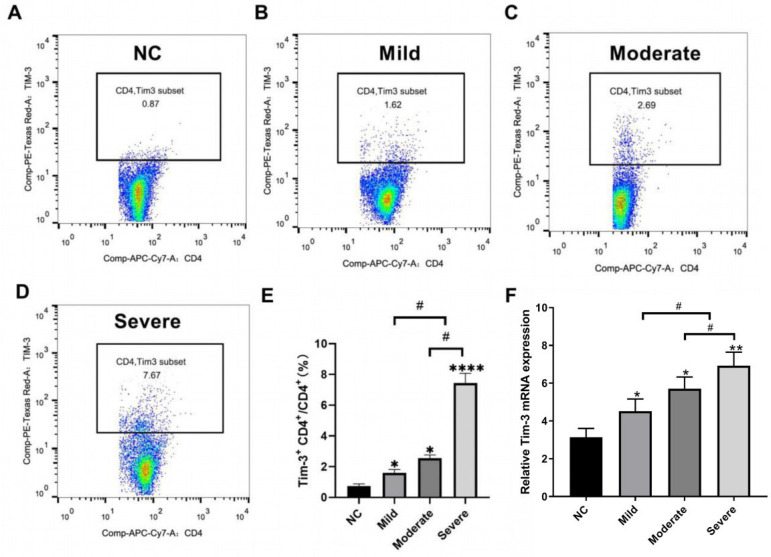
Upregulation of the expression of Tim-3 in CD4+ T cells in peripheral blood of patients with CHD. A-D: Flow cytometry was used to detect the expression of Tim-3 in CD4+ T cells in peripheral blood of the control group, mild group, moderate group and severe group. E: Flow cytometry was used to analyze the quantitative results. F: qRT-PCR was used to detect the expression of Tim-3 mRNA in CD4+ T cells in peripheral blood of each group. *Compared with the control group, *P<0.05, **P<0.01, ****P<0.000; ^#^P<0.05.

We first isolated and purified CD4^+^ T cells from peripheral blood and detected the expression of Tim-3 in CD4^+^ T cells using flow cytometry and qRT-PCR, respectively. The flow cytometry results (Figures 1A to E) demonstrated that the expression of Tim-3 was significantly increased in CD4^+^ T cells in peripheral blood of patients with CHD in mild, moderate and severe groups compared with the control group (*P*<0.05 or *P*<0.0001). The expression of Tim-3 in CD4^+^ T cells in peripheral blood increased as the disease worsened, which meant that the expression of Tim-3 was the highest in the severe group. The results and trends of qRT-PCR were consistent with those of the flow cytometry (Figure 1F), that is, the expression level of Tim-3 in CD4^+^ T cells in peripheral blood increased with aggravation in mild, moderate and severe groups compared with the control group. These results indicated that the expression level of Tim-3 in CD4^+^ T cells in peripheral blood was increased in CHD and was associated with aggravation.

The Level of IL-7 Was Upregulated in Peripheral Blood Serum of Patients with Coronary Heart Disease

**Fig. 2:**
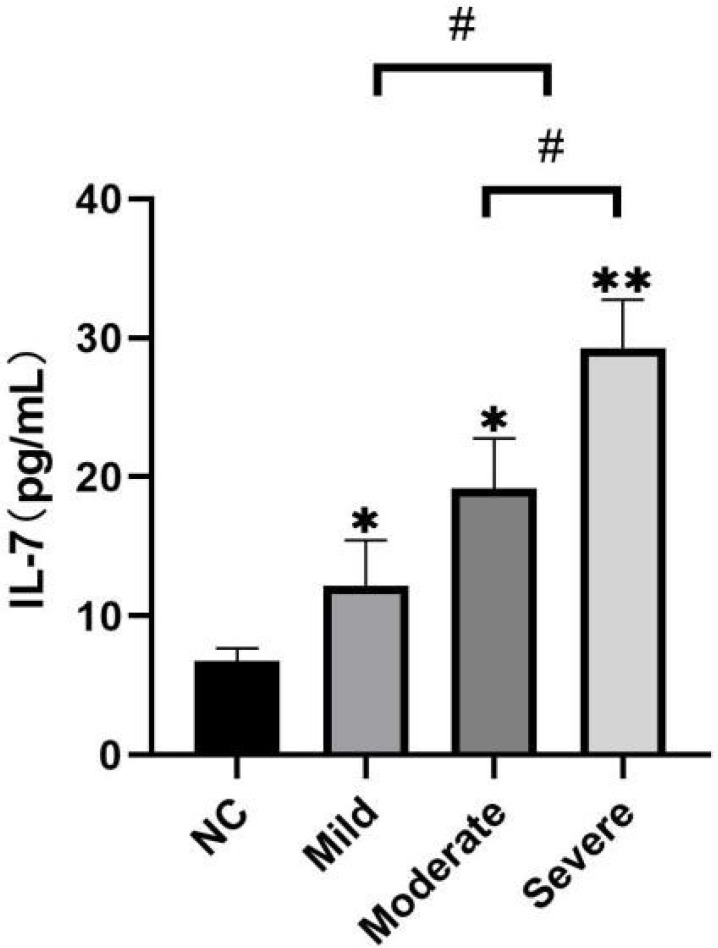
Upregulation of the expression of IL-7 in peripheral blood serum of patients with CHD. The expression level of IL-7 in peripheral blood serum of the study subjects in each group was detected by ELISA. *Compared with the control group, *P<0.05, **P<0.01; ^#^P<0.05.

Next, we detected the expression of IL-7 in peripheral blood serum of study subjects in each group by ELISA. Figure 2 shows that, compared with the control group (6.75±0.94), the expression of IL-7 in the peripheral blood serum of patients with CHD in mild, moderate, and severe groups was significantly upregulated (*P*<0.05 or *P*<0.01). The expression level of IL-7 was upregulated as the disease worsened (*P*<0.05). That is, compared with the mild group, the expression of IL-7 was higher in moderate and severe groups; while compared with the moderate group, the expression level of IL-7 was higher in the severe group. The difference was statistically significant. These results indicated that the expression of IL-7 in peripheral blood serum of patients with CHD was upregulated and associated with the severity of the disease.

### Correlation Analysis of Expression Levels of Tim-3, IL-7 and Severity of Disease

**Fig. 3:**
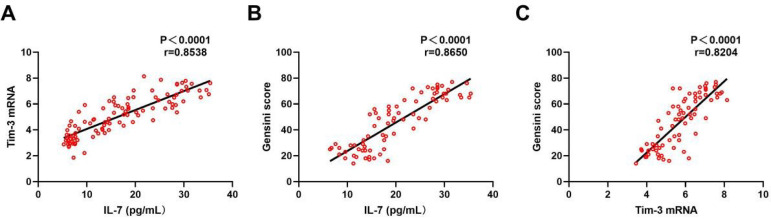
Correlation among the expression levels of Tim-3, IL-7 and the severity of disease. A-C: correlation among Tim-3, IL-7 and Gensini score was analyzed by Pearson correlation test.

The results of the aforementioned studies indicated that the expression levels of both Tim-3 and IL-7 were correlated with disease severity. And the more severe the disease, the higher the expression levels. Therefore, we then used Pearson correlation test to analyze the correlation between the expression of Tim-3 in CD4^+^ T lymphocytes in peripheral blood and the level of IL-7 in peripheral blood serum. At the same time, we respectively analyzed the correlation between these two and the level of Gensini score. As shown in Figure 3, the expression level of mRNA of Tim-3 was positively correlated with IL-7 (*P*<0.0001), which was consistent with the results reported in the literature ^[11]^. Therefore, we inferred that the high expression of Tim-3 in CD4^+^ T lymphocytes in peripheral blood of patients with CHD may be related to the high level of IL-7 in peripheral blood serum. The results in Figure 3B showed that the level of IL-7 in peripheral blood serum of patients with CHD was positively correlated with Gensini score (*P*<0.0001). The results in Figure 3C showed that the expression of Tim-3 in CD4^+^ T lymphocytes in peripheral blood of patients with coronary heart disease was positively correlated with Gensini score (*P*<0.0001), indicating that Tim-3 and IL-7 were indicators of CHD progression. In summary, Tim-3 in CD4^+^ T lymphocytes in peripheral blood of patients with CHD may be associated with elevated IL-7 in peripheral blood serum of these patients, which can be used as a predictor of disease progression in CHD.

## DISCUSSION

It is generally accepted that the occurrence and development of CHD are associated with the immune-inflammatory response. Tim-3 is a member of the TIM gene family. As a negatively regulated immune checkpoint, Tim-3 is expressed in a variety of immune cells *in vivo*, including T cells, dendritic cells, B cells, macrophages, NK and mast cells ^[12]^. Tim-3 has four main ligands, including galectin-9 (Gal-9), carcinoembryonic antigen cell adhesion molecule-1 (CEACAM-1), high mobility group box-1 (HMGB1), and phosphatidylserine (PS). Among them, Tim-3 negatively regulates regulatory T cells by binding to Gal-9, thus suppressing tumor immunity ^[13]^. 

Therefore, Tim-3 has emerged as a potential target for cancer immunotherapy. The immunosuppressive mechanism of Tim-3 has a dual role, which can not only mediate immune tolerance during cancer treatment, but also plays an immunoprotective role in some infectious diseases. Kared et al. ^[14]^ found that, in acute hepatitis C, the signaling pathway mediated by Tim-3 could inhibit IL-21 secreted by Th17 cells and enhance the function of regulatory T cells, thus protecting the body from damage caused by the excessive immune-inflammatory response ^[15]^. 

In this study, we found that Tim-3 was significantly increased in CD4^+^ T cells in peripheral blood of patients with CHD, especially in the severe group, where the expression of Tim-3 was the highest. Combined with the reported literature, we inferred that elevated Tim-3 may negatively regulate the immune-inflammatory response by inhibiting T cell function, thus preventing the myocardium from damage caused by excessive immune attack, which in turn delayed the progression of the disease.

IL-7 is a 25kDa secretory soluble globulin encoded by IL-7 gene, and its receptor (IL-7R) is a heterodimeric complex composed of IL-7Rα (encoded by IL7R) and a common gamma chain (encoded by IL2RG) ^[16]^. 

IL-7 plays a crucial role in regulating the homeostasis of immune cells such as T cells ^[17]^. Zhu et al. ^[18]^ reported that IL-7 induced by *Schistosoma japonicum* infection significantly inhibited macrophage autophagy triggered by schistosome egg antigens, which resulted in liver disease. The study results by Li et al. ^[19]^ suggested that IL-7 could promote the progression of atherosclerosis by activating endothelial cells and monocytes/macrophages through PI3K/AKT-dependent and NF-κB-independent activation. Domås et al. ^[20]^ similarly concluded that IL-7-mediated inflammation promoted the formation of atherosclerosis and led to clinical instability in CHD. The mechanisms involved included interactions between platelets, monocytes, and chemokines. Given that IL-7 has been reported to upregulate the expression of Tim-3 in human T cells ^[11]^, we selected IL-7 in this study to investigate the reasons for the upregulation of Tim-3 in CD4^+^ T cells in peripheral blood of patients with CHD. 

In this study, we found that the IL-7 level in peripheral blood serum of patients with CHD increased with the worsening of the disease, which was consistent with the reported results in the literature. In addition, we also found that the expression of both Tim-3 and IL-7 was positively correlated with the severity of CHD. As mentioned earlier, the upregulation of Tim-3 in infectious diseases can induce immune tolerance, thus protecting the body from excessive attack by the immune system. Combined with the reported literature, we inferred that upregulated IL-7 in peripheral blood serum of patients with CHD could play a protective role by promoting the expression of Tim-3. In summary, IL-7 had a dual effect on coronary atherosclerotic disease, which could promote the formation of atherosclerosis by activating monocytes/macrophages ^[19]^ and platelets ^[20]^, etc., and could also upregulate the level of Tim-3 in CD4^+^ T cells in peripheral blood of patients with CHD. The latter effect dominated the negative regulation of immune-inflammatory response. However, we required further explorations to determine whether IL-7 could positively regulate Tim-3 in CD4^+^ T cells in peripheral blood, whether the function of CD4^+^ T cell in peripheral blood was thereby inhibited, and what roles did IL-7/Tim-3 regulatory axis play in coronary atherosclerotic disease.

## CONCLUSION

In this study, we found for the first time that the expression of Tim-3 was upregulated in CD4^+^ T lymphocytes in peripheral blood of patients with CHD, and the expression of IL-7 was also increased in peripheral blood serum of patients with CHD, which meant they had a positive correlation. Therefore, we inferred that IL-7 may upregulate the expression of Tim-3, and the expression levels of both Tim-3 and IL-7 were positively correlated with the Gensini score. Therefore, we further inferred that Tim-3 and IL-7 can be used as potential biomarkers to predict severity in CHD.

**Table:** 

Authors' roles & responsibilities	
JZ	Substantial contributions to the conception or design of the work; or the acquisition, analysis or interpretation of data for the work; drafting the work or revising it critically for important intellectual content; final approval of the version to be published
FZ	Substantial contributions to the conception or design of the work; or the acquisition, analysis or interpretation of data for the work; drafting the work or revising it critically for important intellectual content; final approval of the version to be published
HL	Substantial contributions to the conception or design of the work; or the acquisition, analysis or interpretation of data for the work; drafting the work or revising it critically for important intellectual content; final approval of the version to be published
